# Novel Human Herpesvirus 8 Subtype D Strains in Vanuatu, Melanesia

**DOI:** 10.3201/eid1311.070636

**Published:** 2007-11

**Authors:** Olivier Cassar, Philippe V. Afonso, Sylviane Bassot, Sabine Plancoulaine, Renan Duprez, Corinne Capuano, Myriam Abel, Paul M.V. Martin, Antoine Gessain

**Affiliations:** * Institut Pasteur de Nouvelle-Calédonie, Nouméa, France; † Institut Pasteur, Paris, France; ‡ Université René-Descartes, Paris, France; § World Health Organization/Organisation Mondiale de la Santé, Port-Vila, Republic of Vanuatu; ¶ Ministry of Health, Port-Vila, Republic of Vanuatu

**Keywords:** Human herpesvirus, HHV-8, Melanesia, molecular epidemiology, Vanuatu, dispatch

## Abstract

We show human herpesvirus 8 with diverse molecular subtype D variants to be highly endemic among the Ni-Vanuatu population. Most K1 genes were nearly identical to Polynesian strains, although a few clustered with Australian or Taiwanese strains. These results suggest diverse origins of the Ni-Vanuatu population and raise questions about the ancient human population movements in Melanesia.

Human herpesvirus 8 (HHV-8), or Kaposi Sarcoma Associated Herpesvirus (KSHV), is the etiologic agent of Kaposi sarcoma (KS). HHV-8 is not a widespread ubiquitous virus; its presence is mainly restricted to areas where classic or endemic KS is highly prevalent, i.e., estimates of HHV-8 seroprevalence in the general adult population range from 5% to >50% (*1*).

Exploiting the highly genetic variability of the HHV-8 K1 gene, molecular epidemiology led to the identification of 5 major K1 subtypes (A–E), some of which appear to be strongly linked to the geographic origin of the samples. Thus, the few known subtype D strains have been reported only in inhabitants from the Western Pacific region (*2*).

For people of Oceanian ancestry (including Melanesian, Polynesian, and Micronesian), very little data are available on the clinical and molecular epidemiology of HHV-8 and its associated diseases (*3**–**9*). Thus, we studied HHV-8 in the Vanuatu, an archipelago in the Southwest Pacific region, formerly named New Hebrides, which contains >80 islands (6 provinces). Indigenous Melanesians, also called Ni-Vanuatu, constitute 98% of the current population of ≈210,000. A recent study suggested that HHV-8 was rare in the Ni-Vanuatu population (*10*). Our goal for this cross-sectional study was to evaluate the prevalence of HHV-8 in the Vanuatu archipelago by using stringent serologic criteria and to characterize its genetic diversity.

## The Study

Our work was performed on a large collection of ≈4,500 plasma and peripheral blood buffy coat (PBBC) samples from different islands of the archipelago, obtained in the framework of our previous studies on human T-cell lymphotropic virus (HTLV-1) (*11**,**12*). The field survey, carried out from April 2003 through August 2005, has been extensively described (*11*).

**Figure 1:**
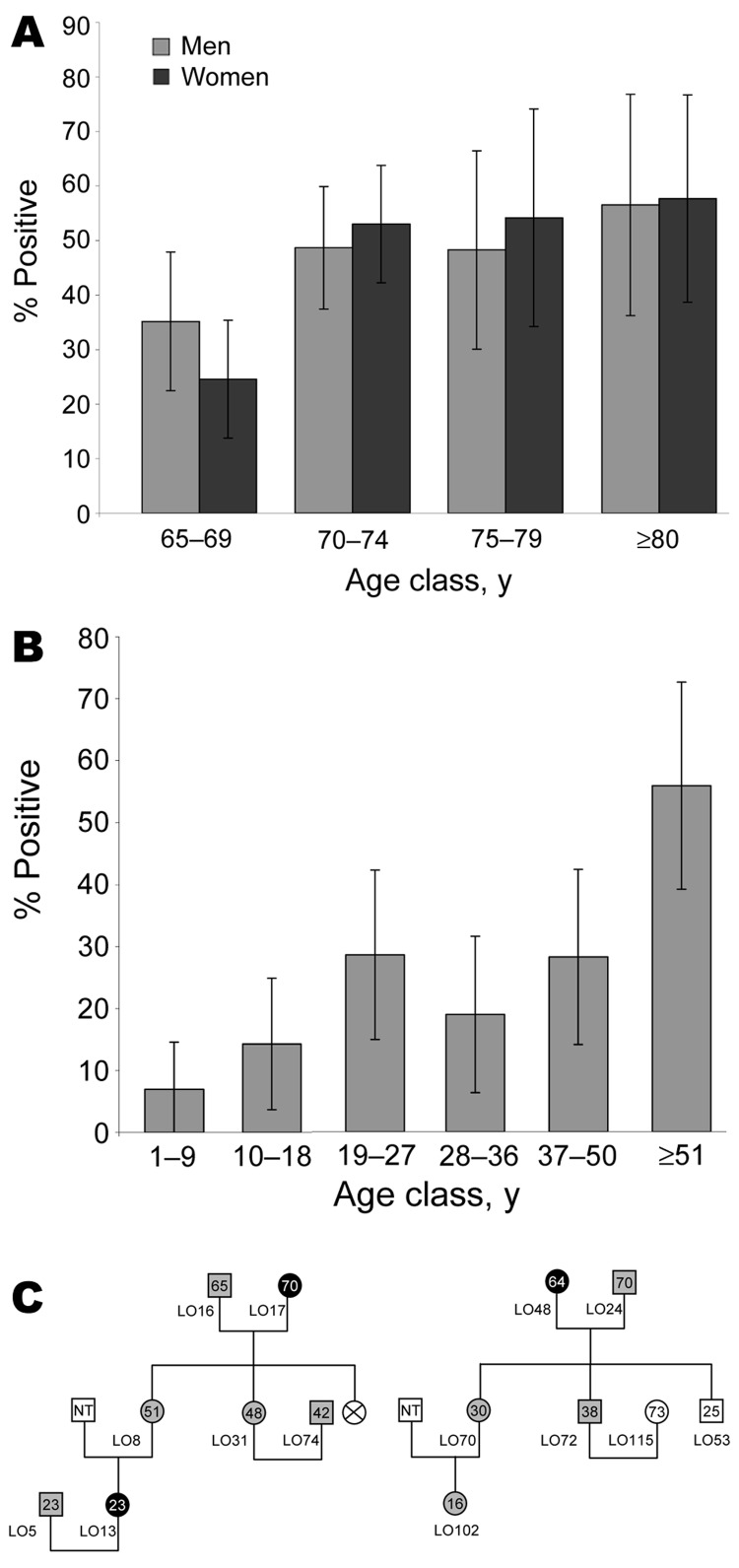
A) Age-dependent herpesvirus 8 (HHV-8) seroprevalence rates in 376 Ni-Vanuatu persons >65 years and living in 18 islands representative of the 6 provinces of the Vanuatu Archipelago. Seropositivity was based on strict criteria, and only samples clearly reactive at a dilution >1:160 were considered HHV-8 positive. B) Age-dependent HHV-8 seroprevalence rate in 283 Ni-Vanuatu persons from 13 families originating from 4 islands (3 from Loh, 2 from Tanna, 4 from Ambae, and 4 from Esperitu Santo) of the Vanuatu archipelago. C) Pedigrees of 2 families from Loh Island in which the presence of HHV-8 was examined in members of 3 generations. Gray circles and squares denote infected women and men, respectively. Black circles denote infected women for whom sequence of K1 gene fragment was obtained. Numbers within circles and squares indicate ages of the patients; NT, not tested.

To detect plasma HHV-8 antibodies, an inhouse immunofluorescence assay (IFA) using BC-3 cells expressing only latent-associated nuclear antigens encoded by ORF73, was performed to detect plasma HHV-8 antibodies (*13*). Because HHV-8 seroprevalence increases with age in a virus-endemic population, we first tested a series of 376 samples, from persons >65 years of age (mean 72, median 70, range 65–96 years; 182 men and 194 women) originating from the 6 provinces of the archipelago (Appendix Figure). Among these 376 plasma samples, 170 (45.2%) were IFA positive at a 1:160 dilution, showing a clear typical nuclear spotted seroreactivity. The HHV-8 seroprevalence was similar between men (45.6%) and women (44.8%). The prevalence of HHV-8 increased with age, rising from 29.6% (65–69 years) to 57.1% (>80 years) (Figure 1, panel A) (p = 0.0005 trend χ^2^ test). This high level of HHV-8 seroprevalence was present in all 6 provinces (Appendix Figure).

A second serologic survey that used 237 plasma samples taken from 13 families with genealogic trees was performed (Figure 1, panels B and C). Among these 237 samples, 12 originated from elderly persons included in the 376 samples tested before. The HHV-8 seroprevalence was clearly age-dependent, rising from 6.9% among children 1–9 years of age to 28.2% in adults 50 years of age, followed by a new increase in persons >51 years of age (55.9%) (p<10^–4^ trend χ^2^ test). These results demonstrate for the first time, to our knowledge, that HHV-8 infection is endemic and circulates in the Ni-Vanuatu population.

We then characterized these HHV-8 strains molecularly. All DNA samples (1 μg), extracted from the PBBC, were first amplified by PCR for human β-globin sequences to control amplifiability. HHV-8 infection was determined by a nested PCR to obtain a 737-bp fragment of the open reading frame of the K1 gene (ORFK1). The first PCR was performed with the primer set K1AG75S/K1AG1200AS (*14*) and followed by a nested PCR with a second set of primers VR1S/VR2AS1 (*15*). All PCR products were purified from gel, cloned, and sequenced. Sequences were verified on both DNA strands. ORFK1 amplification was obtained from 32 (21.6%) of the 148 HHV-8–seropositive samples tested but in none of the 26 HHV-8–seronegative samples. Sequences were obtained for only 30 of the 32 ORFK1-positive PCRs (Table).

**Table:** Demographic, geographic, and serologic data of HHV-8–seropositive persons from the Vanuatu Archipelago, confirmed by molecular analysis*

Virus strain	Age, y	Sex	Island of origin	Province	IFA titers (LANA)	PCR K1	GenBank accession no.
LO 17	70	F	Loh	TORBA	10.240	**+**	EF589758
LO 13	23	F	Loh	TORBA	160	**+**	EF589757
LO 48	64	F	Loh	TORBA	640	**+**	EF589759
ML 10	60	F	Mota Lava	TORBA	640	**+**	EF589763
ML 36	60	M	Mota Lava	TORBA	640	**+**	EF589764
ML 46	61	M	Mota Lava	TORBA	1.280	**+**	EF589765
ES 65	82	F	Santo	SANMA	160	**+**	EF589756
CES W32	60	F	Santo	SANMA	320	**+**	EF589746
MA 55	60	F	Maewo	PENAMA	2,560	**+**	EF589760
MAL 4	74	M	Mallicolo	MALAMPA	320	**+**	EF589761
MAL 24	70	M	Mallicolo	MALAMPA	1.280	**+**	EF589762
RYM 31	80	F	Ambrym	MALAMPA	40	**+**	EF589766
RYM 42	74	F	Ambrym	MALAMPA	80	**+**	EF589767
EP 43	80	F	Epi	SHEFA	20.480	**+**	EF589753
EP 58	79	F	Epi	SHEFA	20.480	**+**	EF589754
EP 111	74	F	Epi	SHEFA	1,280	**+**	EF589755
TON 72	75	M	Tongoa	SHEFA	5.120	**+**	EF589772
CTON H54	71	M	Tongoa	SHEFA	2.560	**+**	EF589747
EM 1	70	M	Emae	SHEFA	640	**+**†	NA
EM 4	75	F	Emae	SHEFA	2.560	**+**	EF589752
CEM H2	73	M	Emae	SHEFA	1.280	**+**	EF589744
EF 43	73	F	Efate	SHEFA	640	**+**	EF589748
EF 52	74	M	Efate	SHEFA	1.280	**+**	EF589749
EF 55	81	M	Efate	SHEFA	640	**+**	EF589750
EF 56	75	F	Efate	SHEFA	5.120	**+**†	NA
EF 60	73	F	Efate	SHEFA	640	**+**	EF589751
CEF H56	72	M	Efate	SHEFA	80	**+**	EF589742
CEF H67	70	M	Efate	SHEFA	640	**+**	EF589743
TA 115	77	M	Tanna	TAFEA	1.280	**+**	EF589768
TA 116	70	F	Tanna	TAFEA	320	**+**	EF589769
TA 124	70	F	Tanna	TAFEA	5.120	**+**	EF589770
TA 162	70	M	Tanna	TAFEA	5.120	**+**	EF589771

*IFA, immunofluorescence assay; LANA, HHV-8–specific antibody directed against latent nuclear antigen; PCR K1, PCR for amplification of a 737-bp fragment of the ORFK1 genomic region of HHV-8; NA, not available. †Weak PCR signal.

Comparative sequence analysis indicates that the 30 new sequences differed from each other. Furthermore, among them, 3 groups can be clearly identified. The first group comprises most strains (23/30) and corresponds to the sequences found in persons from the south central islands of the archipelago (Mallicolo, Ambrym, Epi, Tongoa, Emae, and Tanna); the second group comprises 4 sequences from persons living in the northern islands of Loh (LO13, LO17, LO48) and Santo (CESW32); and the third group involves only 3 sequences (ML10, ML36, ML46) from persons living in the northern island of Motalava.

**Figure 2:**
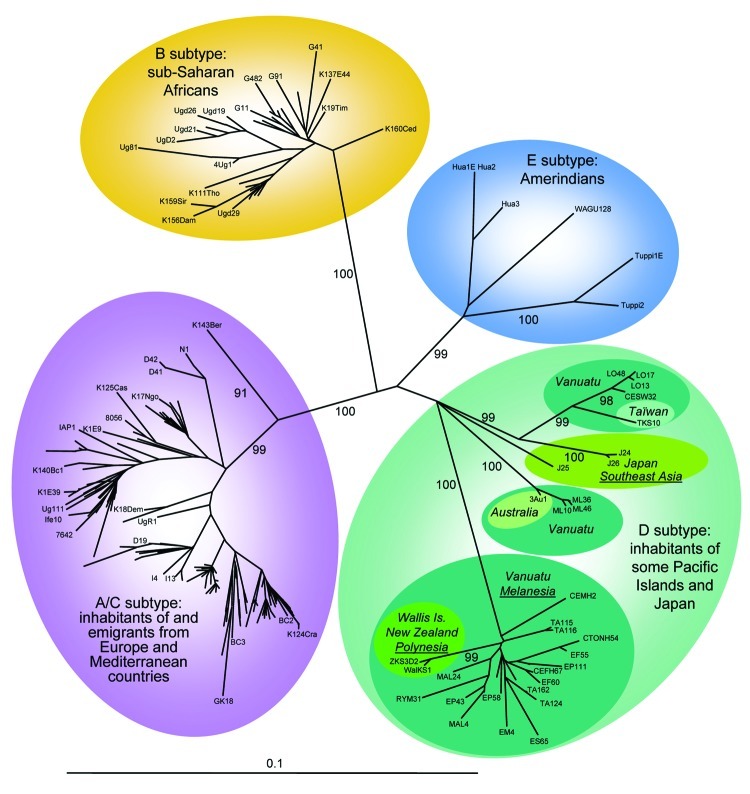
Unrooted phylogenetic tree generated by the neighbor joining (NJ) method with a 624-bp fragment of the K1 gene. The phylogeny was derived by the NJ method by using the GTR model in the PAUP program version 4.0b10 (Sinauer Associates, Sunderland, MA, USA). Reliability of the inferred tree was evaluated by bootstrap analysis on 1,000 replicates. Branch lengths are drawn to scale, with the bar indicating 0.1-nt replacement per site. Numbers on each node indicate the percentage of bootstrap samples (1,000) in which the cluster is supported. Only bootstrap values >75 are given. Not all samples have been labeled because of space constraints. The 30 new ORFK1 HHV-8 sequences (GenBank accession nos. EF589742–EF589771) were analyzed with 195 HHV-8 available sequences from the GenBank database.

Phylogenetic analyses were performed on the 30 novel sequences obtained in this study, on all subtype D available K1 sequences, and on representatives of the different HHV-8 subtypes/subgroups, as described (*4*). The phylogenetic analyses were performed with all of the sequences available to date in GenBank. These sequences include 3 strains from Japan (J24, J25, and J26), 1 from Australia (3Au1), 1 from Taiwan (TKS10), 1 from New Zealand (ZKS3), and 1 from Wallis (WalKS1) (*2**,**4**, **6**–**8*). Our results demonstrate that the Ni-Vanuatu HHV-8 clustered in 3 different genotype D subclades, which are highly supported phylogenetically with high bootstrap values of 99% or 100% (Figure 2). The first one comprising most strains corresponds to sequences closely related to each other and to the 2 Polynesian strains WalKS1 and ZKS3 (*4**,**8*). The second group (Loh/Santo), with only 4 sequences, was closely related to the Taiwanese strain TKS10 (*8*). The last 3 sequences from Motalava were nearly identical to the only strain from Australia (*6*).

Furthermore, phylogenetic analysis showed a star-like tree with a long branch for the Polynesian clade, which includes most Ni-Vanuatu strains. This finding strongly suggests a common origin or ancestor for these strains, with a possible founder effect (Figure 2). Based on stringent serologic and molecular analyses, our study demonstrates for the first time, to our knowledge, that HHV-8 infection is endemic in a Melanesian population.

Our serologic findings are consistent with those found in some remote villages of Papua New Guinea (*5**,**9*). On the basis of these studies, it is tempting to suggest that intrafamilial HHV-8 transmission occurs in such populations, as previously demonstrated in highly HHV-8 endemic populations of African origin (*13*).

From the molecular point of view, finding such a high molecular diversity of HHV-8 subtype D with some Polynesian-, Taiwanese- and Australian-like strains was surprising. These heterogeneous findings contrast with the more homogenous situation found for HTLV-1 genotypes in the same population (*11*).

## Conclusions

Our molecular findings suggest that HHV-8 has been introduced in the Ni-Vanuatu populations by different migrations of infected persons. This conclusion is strengthened by the clustering of the Australian- and Taiwanese-like strains in the northern islands of Loh and Motalava. A variety of scenarios have been proposed to explain the peopling of near and remote Oceania, and our data highlight the possible multiple origins of Ni-Vanuatu ancestors. Ongoing molecular studies on both viral and mitochondrial/nuclear DNA will contribute to this debate through analyses of the variations observed. Indeed, these variations are intimately linked with the dispersal of early human settlers; analyses of the genetic variability of HHV-8 can help us reconstruct the patterns of human dispersal into Oceania (*11*).

## Supplementary Material

Appendix Figure Map of Vanuatu Archipelago showing the distribution of human herpesvirus 8 (HHV-8) seroprevalence in persons >65 years of age and living in different provinces. The 6 administrative divisions studied were the Torba Province, comprising mainly Torres and Banks Islands; the Sanma Province, comprising Esperitu Santo and Malo Islands; the Penama Province, comprising Pentecost, Ambae, and Maewo Islands; the Malampa Province, comprising Malekula, Ambrym, and Paama Islands; the Shefa Province, comprising mainly Shepherds and Efate Islands; and the Tafea Province, comprising Tanna, Erromango, and Aneityum Islands. For each area, the number of persons tested and the number and percentage (in parentheses) of HHV-8-seropositive samples (immunofluorescence assay for latent nuclear antigens) are indicated. The mean age and the gender of the studied population are also shown. To have a good specificity, we considered as HHV-8 positive only samples that were clearly reactive at a dilution >1:160.
