# Supplementary figures and images for: Novel Human Herpesvirus 8 Subtype D Strains in Vanuatu, Melanesia
Source: Emerg Infect Dis. 2007 Nov;13(11):1745–8. doi: 10.3201/eid1311.070636 (PMC3375799; doi:10.3201/eid1311.070636)

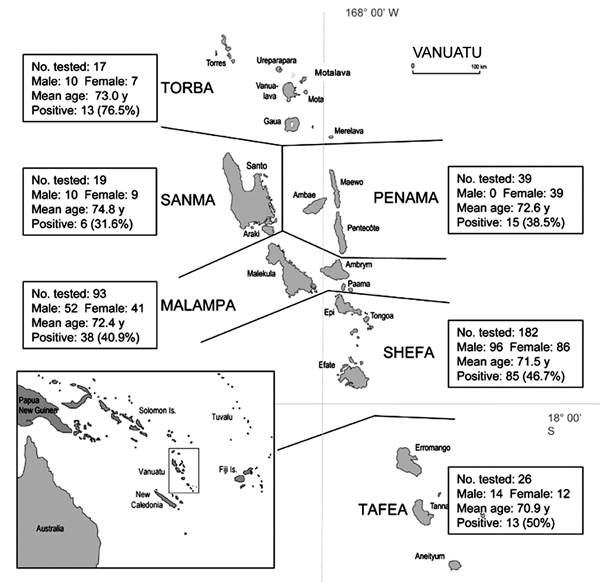

Supplement: Appendix Figure — Map of Vanuatu Archipelago showing the distribution of human herpesvirus 8 (HHV-8) seroprevalence in persons >65 years of age and living in different provinces. The 6 administrative divisions studied were the Torba Province, comprising mainly Torres and Banks Islands; the Sanma Province, comprising Esperitu Santo and Malo Islands; the Penama Province, comprising Pentecost, Ambae, and Maewo Islands; the Malampa Province, comprising Malekula, Ambrym, and Paama Islands; the Shefa Province, comprising mainly Shepherds and Efate Islands; and the Tafea Province, comprising Tanna, Erromango, and Aneityum Islands. For each area, the number of persons tested and the number and percentage (in parentheses) of HHV-8-seropositive samples (immunofluorescence assay for latent nuclear antigens) are indicated. The mean age and the gender of the studied population are also shown. To have a good specificity, we considered as HHV-8 positive only samples that were clearly reactive at a dilution >1:160. [file 07-0636_appF-s1.gif]
